# Real-Time Sensing of Cell Morphology by Infrared Waveguide Spectroscopy

**DOI:** 10.1371/journal.pone.0048454

**Published:** 2012-10-31

**Authors:** Victor Yashunsky, Tal Marciano, Vladislav Lirtsman, Michael Golosovsky, Dan Davidov, Benjamin Aroeti

**Affiliations:** 1 The Racah Institute of Physics, The Hebrew University of Jerusalem, Jerusalem, Israel; 2 Department of Cell and Developmental Biology, The Alexander Silberman Institute of Life Science, The Hebrew University of Jerusalem, Jerusalem, Israel; Tel Aviv University, Israel

## Abstract

We demonstrate that a live epithelial cell monolayer can act as a planar waveguide. Our infrared reflectivity measurements show that highly differentiated simple epithelial cells, which maintain tight intercellular connectivity, support efficient waveguiding of the infrared light in the spectral region of 1.4–2.5 µm and 3.5–4 µm. The wavelength and the magnitude of the waveguide mode resonances disclose quantitative dynamic information on cell height and cell-cell connectivity. To demonstrate this we show two experiments. In the first one we trace in real-time the kinetics of the disruption of cell-cell contacts induced by calcium depletion. In the second one we show that cell treatment with the PI3-kinase inhibitor LY294002 results in a progressive decrease in cell height without affecting intercellular connectivity. Our data suggest that infrared waveguide spectroscopy can be used as a novel bio-sensing approach for studying the morphology of epithelial cell sheets in real-time, label-free manner and with high spatial-temporal resolution.

## Introduction

Living cells can act as optical devices. For instance, recent studies have demonstrated single-cell lasing [1] and optical-fiber-like functioning of the Muller retinal cells [2]. In this work we show that a live epithelial cell monolayer can operate as a planar optical waveguide in the infrared spectral region. Several factors conspire to enable the propagation of infrared waveguide modes in cell layers: (i) the ability of cells to self-assemble and form a tightly-bound monolayer; (ii) the refractive index of cells is higher than that of the surrounding aqueous media; (iii) the cell height is on the order of the infrared wavelength. We succeeded to excite waveguide modes in different epithelial cell types using prism coupler and collimated infrared light. The waveguide mode excitation is associated with resonant reflectivity minima at certain incident angles. The magnitude and wavelength of these resonances is determined by the intercellular connectivity and cell monolayer thickness (i.e., the average cell height).

These waveguide mode resonances are sensitive to cell monolayer structure that is controlled by interactions between the cell cytoskeleton, the membrane, membrane-bound proteins, and the extracellular environment [3] and reports on the state of the cell monolayer in the same way as the structure of an individual cell manifests it’s functioning [4]. While convenient means to study cell structure such as confocal [5] and atomic force microscopy [6], [7], are excellent for accurate measurements of structural changes in single cells but have decreased spatial and temporal resolution in monitoring of cell monolayer structure. The waveguide spectroscopy method that we propose here is capable to quantitatively assess the cell monolayer structure containing large cell population with temporal resolution of a few seconds and submicron resolution in cell monolayer height. We demonstrate two case studies in which we use the waveguide mode spectroscopy to track the cell monolayer structure upon Ca^2+^ switch and PI3-kinase inhibition.

## Results

### Wave Propagation in a Live Cell Monolayer Cultured on Substrate

**Figure 1:**
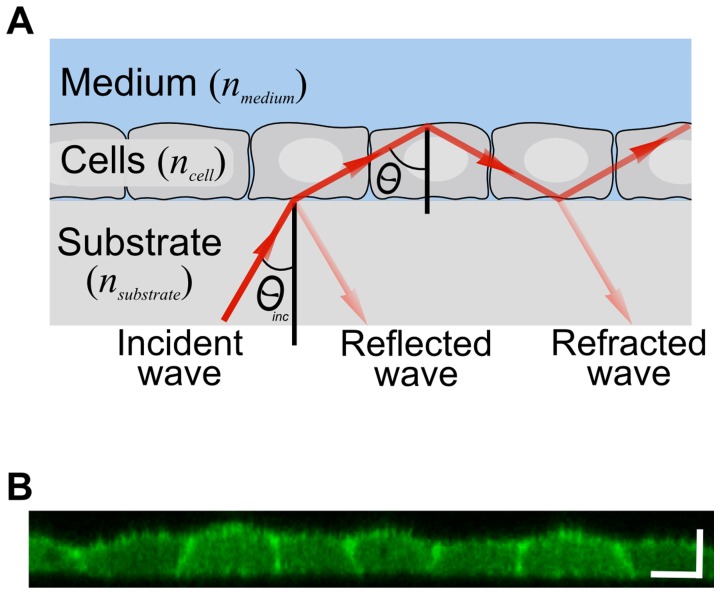
Schematic representation of intracellular leaky waveguide mode propagation in a living cell monolayer. A. Waveguide mode excitation in a living cell monolayer. An electromagnetic wave penetrates at an incident angle *θ_inc_* from the high-refractive-index substrate into a cell monolayer having a lower refractive index, *n_cell_*_,_. Because *n_medium_* is lower than *n_cell_*_,_ this wave undergoes total internal reflection at the cell-medium interface. The wave then impinges on the cell-substrate interface where it is partially reflected (solid red arrow) and refracted (pale red arrow). Excitation of the radiative (leaky) waveguide mode occurs when the reflected and refracted waves at the substrate-cell interface interfere destructively, confining the energy within the cell layer. B. XZ-section of epithelial MDCK cell monolayer stably expressing LifeAct-GFP as imaged by confocal microscopy. Scale bars: 10 µm.

**Figure 2:**
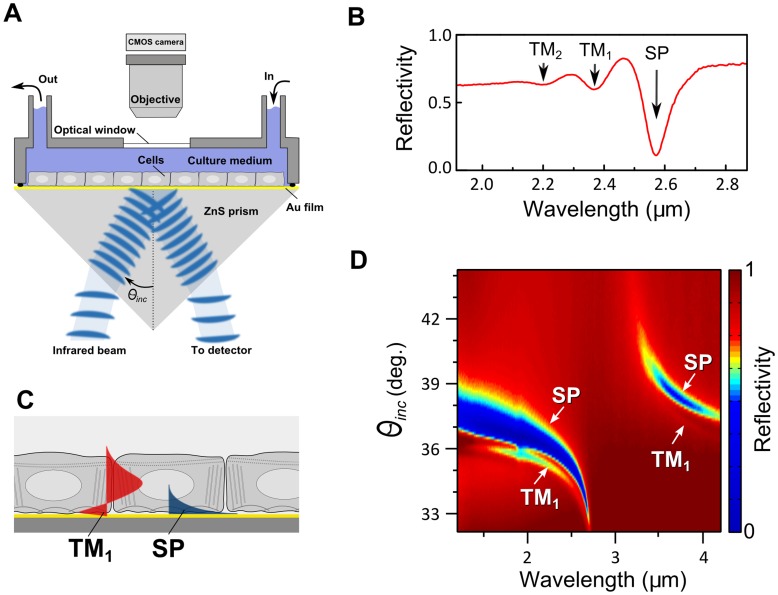
Excitation of intracellular waveguide modes using a collimated broadband infrared beam. A. Experimental setup. A cell layer was cultured on an Au-coated ZnS prism as described in Materials and Methods. During the measurement, the cells in the flow chamber were exposed to culture medium at constant flow. The collimated and polarized infrared beam from the FTIR spectrometer impinges on the gold layer at angle *θ_inc_* and excites waveguide modes within the cell layer (panel C). The intensity of the reflected beam is measured by an MCT detector. Simultaneously, the cells are optically imaged by a CMOS camera attached to the optical microscope. B. Wavelength-dependent reflectivity measurement (*I_p_/I_s_*, see Materials and Methods) from the ZnS/Au/MDCK cells/medium assembly at *θ_inc_* = 34.8°. The reflection minima correspond to the surface plasmon (SP) resonance and to the waveguide mode resonances (TM_1_ and TM_2_). C. Schematic representation of the electric field distribution for the surface plasmon and TM_1_ waveguide mode propagating in the cell layer. The surface plasmon penetrates only up to ∼2 µm into the cell layer and is thus sensitive mainly to the cell-substrate interface. The TM_1_ mode penetrates much further, it is confined within the entire cell volume and can be used to measure the cell height *h*. D. Angular-resolved reflectivity spectra from the ZnS/Au/MDCK cells/medium assembly. The strong reflectivity minimum (deep blue) arises from the surface plasmon resonance. Its angular dependence mimics the dispersion of the water refractive index. A shallow minimum at lower angles (light blue) corresponds to the TM_1_ waveguide mode. This mode does not appear in the absence of cell layer (i.e., in a bare Au substrate; see Figure S1).

We noticed that the cell monolayer cultured on a substrate can be considered as a planar optical waveguide. From a “physicist’s point of view”, a ∼10 µ*m*-thick cell monolayer tightly attached to flat substrate and bathed with growth medium (99% water) forms a dielectric trilayer with progressively decreasing refractive indices, n_substrate_>n_cell_>n_medium_ (Figure 1). When the angle of incidence at the cell-medium interface exceeds the critical angle (∼*78*°) at the cell-medium interface the cell monolayer can support leaky waveguide modes also known as radiative modes [8], [9]. Prism or grating can be used to resonantly couple light into the waveguiding trilayer and to excite these modes (Figure 2A).

The waveguide resonance occurs when the total phase shift for round-trip propagation is an integral multiple of *2π*. Namely, *2φ+φ_cm_+φ_cs_ = 2mπ,* where *φ* = *n_cell_k_0_h*cos*θ* is the phase shift on wave propagation through the cell layer, and *φ_cs_*, *φ_cm_* are phase shifts on reflection from the cell-medium and cell-substrate interfaces, respectively. Here, *k_0_ = 2π/λ* is the incident wave vector and *h* is the cell layer thickness. For *p*-polarized incident light these relations yield a set of resonant wavelengths corresponding to TM_1_, TM_2_,… modes,
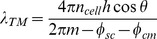
(1)where *m* = 1,2,… The resonant wavelengths can be tuned by varying the angle *θ*.

The reflectivity from the trilayer assembly is given by the Airy formula [10]

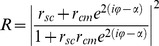
(2)where *r_sc_* and *r_cm_* are complex Fresnel reflection coefficients at the substrate-cell and cell-medium interfaces, correspondingly; *α = µ_cell_h/*cos*θ* is the total attenuation during round trip propagation in the cell layer and *µ_cell_* is the attenuation in the cell layer per unit length. At certain wavelengths/angles the reflectivity *R* achieves its minimum due to destructive interference of the waves reflected from the substrate-cell and cell-medium interfaces (reflected and refracted waves; Figure 1A). This minimum corresponds to the waveguide mode excitation. The minimal reflectivity, Δ*R_TM_ = R_off-resonance_−R_resonance_*, is determined by the wave attenuation, *α*. Apart from the intracellular absorption and scattering, there can be dynamic losses associated with the incomplete cell-cell attachment. This allows us to exploit the magnitude of the waveguide resonance Δ*R*_TM_ as an indicator of dynamic changes in the cell-cell connectivity.

The reflection from the cell-substrate interface can be significantly improved by introducing a thin conducting layer on top of the substrate. This has some effect on the resonant wavelength *λ_TM_* since the phase shift at the cell-substrate interface becomes *φ_cs_≈π* (Eq. 2). In addition, conducting layer enables excitation of the TM_0_ mode which is essentially the surface plasmon (SP) [11], [12]. It should be noted that the SP mode in cell layer is different from the waveguide modes in two aspects. First, the SP mode does not require continuous cell monolayer and can be excited in disconnected cells as well. The SP resonant wavelength is
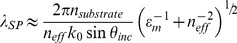
(3)Here, *ε_m_* is the real part of the metal dielectric constant, *n_eff_ = (1*−*f)n_medium_+fn_cell_* is the effective refractive index of the cell layer, and *f* is the cell coverage. Second, the SP field exponentially decays away from the conducting film and does not reach the cell-medium interface, thus its resonant wavelength *λ_SP_* practically does not depend on cell height [13], [14].

In one experiment we can measure the waveguide and the surface plasmon resonance that yield complementary information on cell layer. The *λ_TM_* and Δ*R_TM_* measure the average cell height and the degree of intercellular attachment, while *λ_SP_* monitors the cell-substrate coverage.

### Waveguide Mode Excitation in a Live Cell Monolayer

We reasoned that a tight epithelial cell monolayer could serve as a suitable model to observe the waveguide modes in live cells. We used the Madin-Darby canine kidney (MDCK type II) cells which are non-cancerous and highly differentiated renal epithelial cells, commonly used to study epithelial cell biology and epithelial tissue development [3]. These cells typically grow as a tight and continuous monolayer with an average height of *h* = 8-to-14 µm, depending upon cell culturing conditions, and have intra-layer height variation of only Δ*h*∼1 µm [15]–[17]. A typical cross-section of GFP-LifeAct-expressing MDCK cell monolayer visualized by confocal microscopy is presented in Figure 1B.

We cultured MDCK cells as a confluent monolayer for three days directly on an Au-coated ZnS prism (see Figure 2A and Materials and Methods) and measured the reflectivity spectra at oblique angle using a Fourier-Transform Infrared (FTIR) spectrometer [18]. At certain wavelengths we observed reflectivity minima which we attributed to the surface plasmon (SP) and to the waveguide mode resonances (TM_1_ and TM_2_; Figure 2B). While the SP appears also in the absence of cells, the TM-resonances appear only in the presence of intact cell monolayer (Figure S1). This interpretation is also supported by computer simulation based on Fresnel reflectivity formulae for a quad-layer (ZnS/Au/cells/medium) with cell height *h* as a fitting parameter [19]–[21] (Figure S2).

The TM_1_-mode has been also observed in cancerous and poorly differentiated epithelial human cells (e.g., the melanoma MEL 1106, and the cervix carcinoma HeLa cells (Figures S3A-D). Interestingly, however, the TM_1_-mode resonance was deeper in the non-cancerous MDCK (Figures S3E and F) and IEC6 (not shown) epithelial cells. The TM_2_-mode has been observed so far only in the non-cancerous cell layers. These data suggest that tightly interconnected and polarized epithelial cell monolayers support waveguide mode propagation.

### Waveguide Modes Emerge only When Cells Develop Intercellular Contacts

To prove that the observed resonances are related to waveguide mode excitation in the cell layer rather than in individual cells, we studied dynamics of these resonances during cell monolayer formation. The suspended MDCK cells were introduced into the flow chamber and allowed to adhere to the Au-substrate until a confluent cell monolayer is formed. The MDCK cell monolayer formation exhibits three phases [18] (Figure S4): (I) an early phase of cell-substrate attachment and spreading; (II) an intermediate phase whereby cell-cell contacts are established and the small cell clusters appear; (III) a late phase at which voids between cell clusters are closed (healed) until a tight and fully confluent cell monolayer is formed.

**Figure 3:**
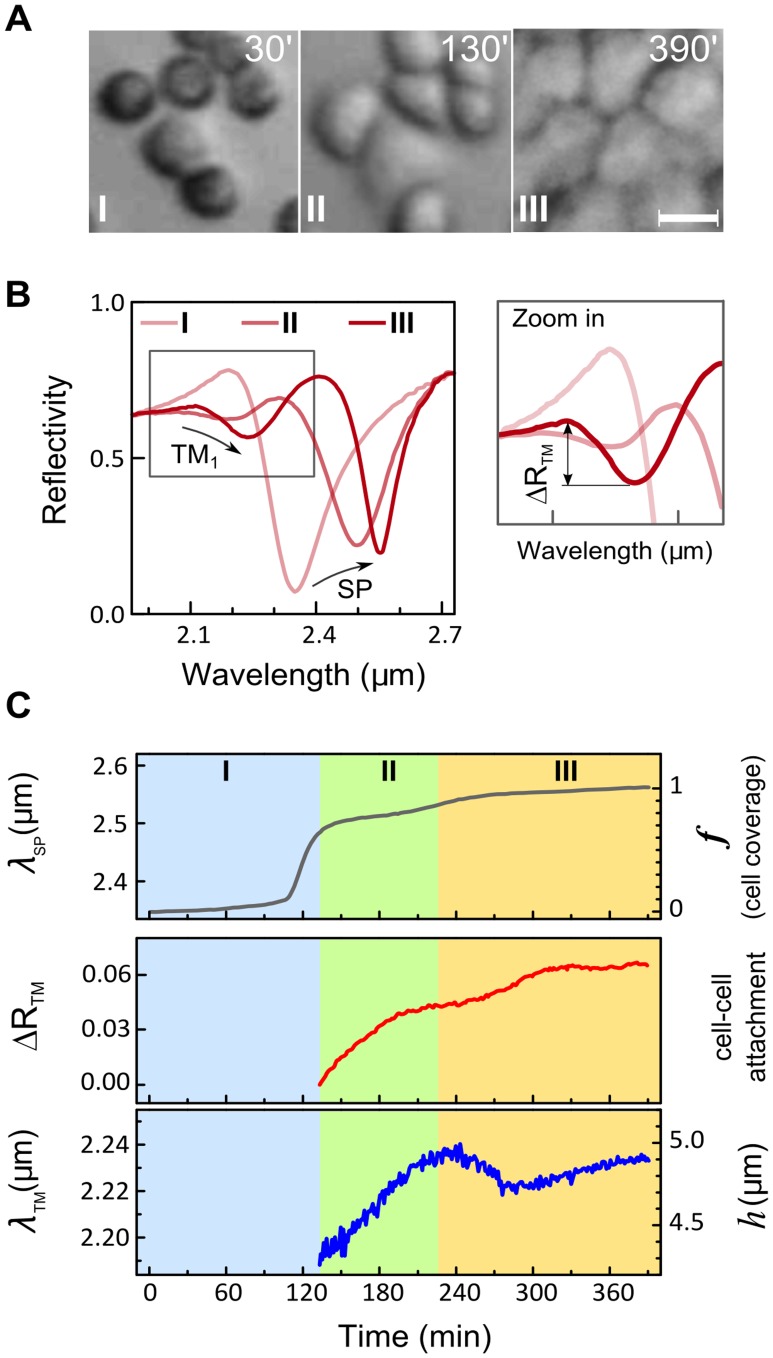
Waveguide mode appears only when intercellular contacts are formed. A. Three phases in MDCK cell monolayer formation as visualized by optical microscopy. Suspended MDCK cells were introduced and allowed to adhere to the gold substrate. Scale bar = 20 µm. B. Infrared reflectivity spectra measured during the three phases of monolayer formation. The surface plasmon resonance shifts towards longer wavelengths as cells attach and cover the Au substrate. The TM_1_ waveguide mode appears only at phase II, in parallel to the establishment of intercellular contacts by neighboring cells. The increasing magnitude of the TM_1_ resonance, *ΔR_TM_*, is associated with the development and tightening of cell-cell contacts. The progressive red-shift of the resonant wavelength *λ_TM_* results from the increasing cell height. C. Time-resolved measurements of *λ_SP_*, *ΔR_TM_*, and *λ_TM_*. The right *y*-axes show the corresponding changes in the cell coverage *f*, cell-cell attachment and cell height *h*. The distinct phases in monolayer formation (I, II and III) are indicated.

Cell monolayer formation was tracked simultaneously by optical microscopy (Figure 3A) and by the infrared reflectivity spectral measurements (Figure 3B) at 1 min time intervals. While the surface plasmon wavelength *λ_SP_* progressively increases during all three phases due to growing cell coverage (Figure 3C, upper panel), the TM_1_ waveguide mode (Figure 3C, middle and lower panels) appears only at phase II, upon the initiation of cell-cell contacts and formation of intact cell clusters. The magnitude of the TM_1_ resonance is influenced by the quality of the intercellular contacts. Thus, the establishment of cell-cell contacts during phase II is the main contributing factor to the increase in Δ*R_TM_*. Further increase in Δ*R_TM_* during phase III suggests development of tighter cell-cell contacts in the cell monolayer.

The resonance wavelength *λ_TM_*, which measures the average cell height in a continuous cell monolayer (see Eq. 1), increases during phase II. This suggests that the height of the cells, which attach to each other, grows. Immediately thereafter, at the beginning of phase III (230–280 min), *λ_TM_* temporarily decreases. This indicates the cell height decrease which is probably associated with cell flattening during cluster merging and void closure. A similar decrease in cell height has been reported for cells lining the leading edge of a wounded cell monolayer in wound-healing experiments [17]. Following this phase, the cell height slowly grows again, and after 48 hrs of incubation it reaches *h*∼8 µm.

This experiment proves that the waveguide mode appears only in the well-connected cell monolayer and that the resonance wavelength can monitor the average cell monolayer height.

### Waveguide Modes Sense Changes in Cell Monolayer Integrity in Response to Calcium Depletion and Replenishment

**Figure 4:**
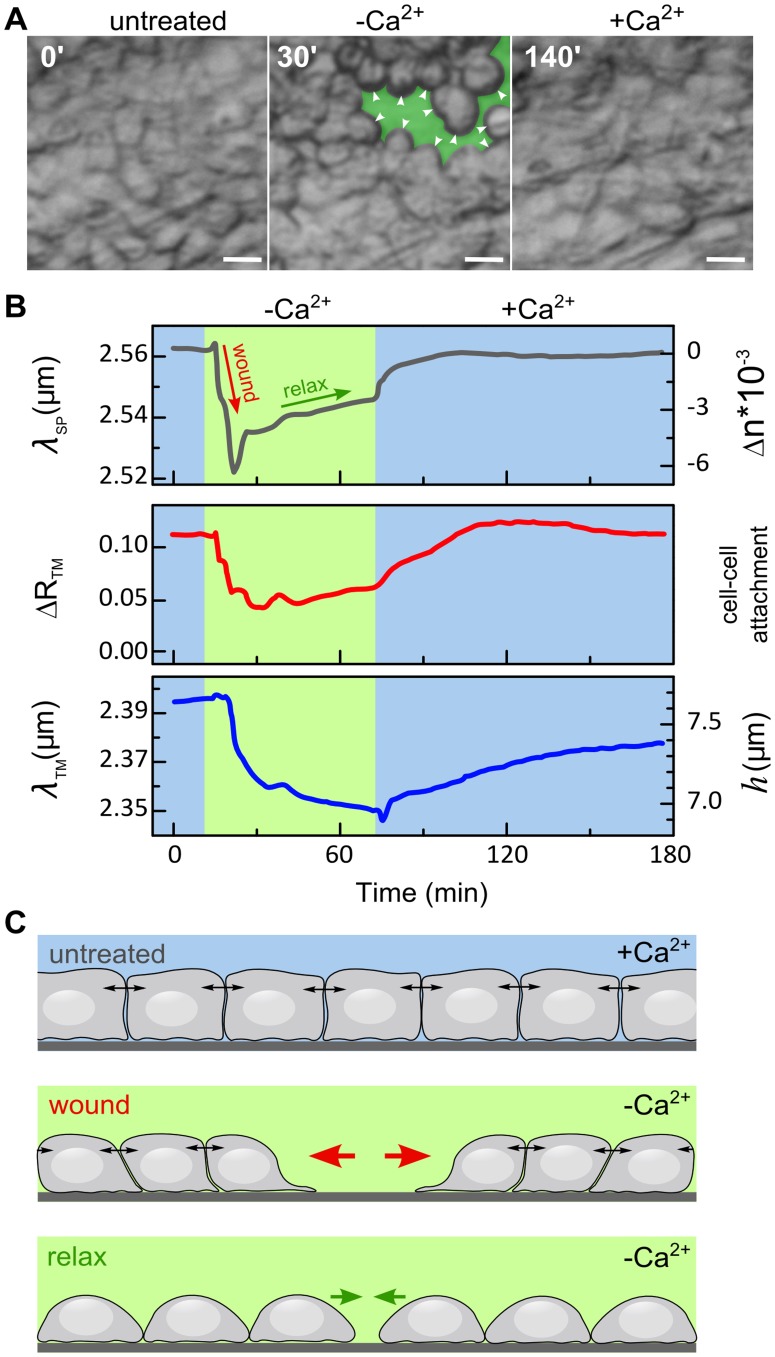
The effects of extracellular Ca^2+^ concentration on MDCK cell monolayer structure. A. Optical microscopy. MDCK cells were cultured as a confluent monolayer in Ca^2+^-containing growth medium for 3 days (untreated). The same cells were then exposed to low-Ca^2+^ medium for 60 min (−Ca^2+^). The appearance of cell-free areas (wounds) in the Au substrate in response to exposure to low-Ca^2+^ medium are artificially colored in green. The cells that encircle the wounded areas achieve a round shape (white arrowheads). The cell layer was then exposed to the same medium supplemented with physiological Ca^2+^ concentrations (+Ca^2+^), that corresponds to conditions under which the wounds in the cell layer heal. Scale bar: 20 µm. B. Time-resolved measurements of *λ_SP_*, *ΔR_TM_* and *λ_TM_*. Initially, the cells were maintained in Ca^2+^-containing growth medium, and measurements were taken continuously for 15 min (blue). Then, low-Ca^2+^ medium was introduced to the flow chamber for approximately 60 min (−Ca^2+^; green panel). Thereafter, cells were re-exposed to the Ca^2+^-containing medium. The dynamic response to cell monolayer wounding (red arrow) and relaxation (green arrow) in low-Ca^2+^ medium are discussed below and in the text. C. Schematic model for the cell monolayer response to low-Ca^2+^ treatment. Upper panel: In the presence of Ca^2+^, namely, prior to Ca^2+^ depletion (untreated) or after Ca^2+^ replenishment (+Ca^2+^), the cells form an intact monolayer and maintain tight intercellular cell-cell junctions. These junctions, which are known to be tightly connected to the cortical actin cytoskeleton, produce pulling forces on neighboring cells (indicated by double-headed arrows, see also [24]). Middle panel: the lowering the Ca^2+^ concentration results in the appearance of cell-devoid areas (wounds) in the monolayer. These areas are expanded by pulling forces applied by the cells in the intact monolayer surrounding the wounded area (red arrows). Lower panel: At later stages and under low Ca^2+^ conditions, cell-cell junctions are progressively damaged, leading to the relaxation of the intercellular cortical tension. Since cell-substrate adhesion is Ca^2+^-insensitive [25], the relaxation of intercellular cortical tension allows cell spreading and thereby partial recovery of substrate coverage.

In the next experiment we analyzed the response of an intact MDCK monolayer to morphological perturbations induced by alterations in extracellular Ca^2+^ concentration. Exposure of an intact epithelial cell monolayer to low-calcium medium results in disruption of cell-cell adhesion and in cell morphology changes. The effect is reversible, the Ca^2+^ replenishment restores the cell-cell contacts [22] (Figure 4A). We expected that the disruption of cell monolayer integrity by Ca^2+^ depletion and its restoration by Ca^2+^-replenishment could be tracked by observing the wavelength and magnitude of the waveguide and SP resonances. Indeed, exposure of the cell monolayer to low-calcium medium led to a sharp blue-shift in *λ_SP_* (Figure 4B, upper panel), corresponding to decreasing cell coverage. This response was consistent with optical images where we observed the appearance of wounds (i.e., cell-devoid regions) on the substrate following Ca^2+^ depletion (see green colored area in Figure 4A, middle panel). Surprisingly however, ∼15 min after cell exposure to low Ca^2+^ medium, *λ_SP_* was red-shifted, indicating that cell coverage was partially restored. This phenomenon could be attributed to a reduction in cortical actin tension (further explained below). Cell exposure to medium containing normal Ca^2+^ concentration (+Ca^2+^), produced a red-shift in *λ_SP_*, which eventually came back to the original pretreatment value. Correspondingly, the gaps in cell monolayer were completely healed (Figure 4A, 140 min). Similar to *λ_SP_*, Δ*R_TM_* and *λ_TM_* (Figure 4B, middle and lower panels, respectively) revealed a reversible dynamic profile, but with slower response.

We suggest the following model to explain these data. Prior to Ca^2+^ depletion, MDCK cells were cultured in medium containing normal Ca^2+^ level, conditions under which the cells form a confluent monolayer with tight cell-cell and cell-substrate adherent junctions. As previously described [17], [23], [24], the existence of actomyosin-bound intercellular junctions (e.g., tight and adherence junctions) generate a tensed cell layer due to pulling forces on the cell cortex (periphery) (exemplified by double arrowheads in Figure 4C). Ca^2+^ depletion disrupts cell-cell junctions [25], resulting in local loss of cell-cell, but not cell-substrate contacts. Then, due to the imposed pulling forces between contacting cells, these “localized wounds” further expand and cell-free substrate regions appear (red arrows in “wound”; Figure 4C and green-colored areas; Figure 4A, 30 min). This phase corresponds to the fast blue-sift in *λ_SP_* (“wound”; Figure 4B, upper panel). Continuous cell exposure to low-Ca^2+^ medium results in progressive disassembly of intercellular junctions and in lowering of the intercellular tension (“relax”; Figure 4C). Disruption of cell-cell contacts contributes to the decrease of Δ*R_TM_*. At the same time, the lowering of tension results in cell height reduction which is detected by a blue-shift in *λ_TM_*. Since the cell-substrate adhesion is mediated by Ca-insensitive integrins and Ig-superfamily adhesion molecules [25], cell coverage could be partially restored due to relaxation of the intercellular tension (Figure 4C, “relax”), as indicated by the gradual red-shift in *λ_SP_* (Figure 4B, upper panel). Ca^2+^ replenishment causes a fast (20 min) and full recovery in cell coverage, as indeed indicated by the sharp red shift in *λ_SP_* (Figure 4B, upper panel). The restoration of cell-cell contacts is slower (45 min). The *ΔR_TM_* recovers accordingly (Figure 4B, middle panel). Cell height restoration, measured by a red shift in *λ_TM_*, occurs even at a slower pace (Figure 4B, lower panel). This reflects the longer time needed for the establishment of cell-cell junctions mediating the actomyosin tension required for columnar cell shaping [26].

### Phosphatidylinositol 3-kinase (PI3K) Inhibition Reduces Cell Height Without Affecting Epithelial Monolayer Intactness

The treatments applied so far are known to have concomitant effects on cell height, cell-cell and cell-substrate contacts. Our next aim in the context of examining the analytical abilities of the waveguide spectroscopy was to apply a treatment that will have a selective effect. For instance, we looked for a treatment that would affect the cell height, but not the monolayer integrity. Indeed, confocal microscopy studies on fixed MDCK cell have shown that inhibition of the PI3K with LY294002 (LY) has no detectable effect on MDCK monolayer intactness, while in all cases it significantly reduced the cell height [15], [16].

**Figure 5:**
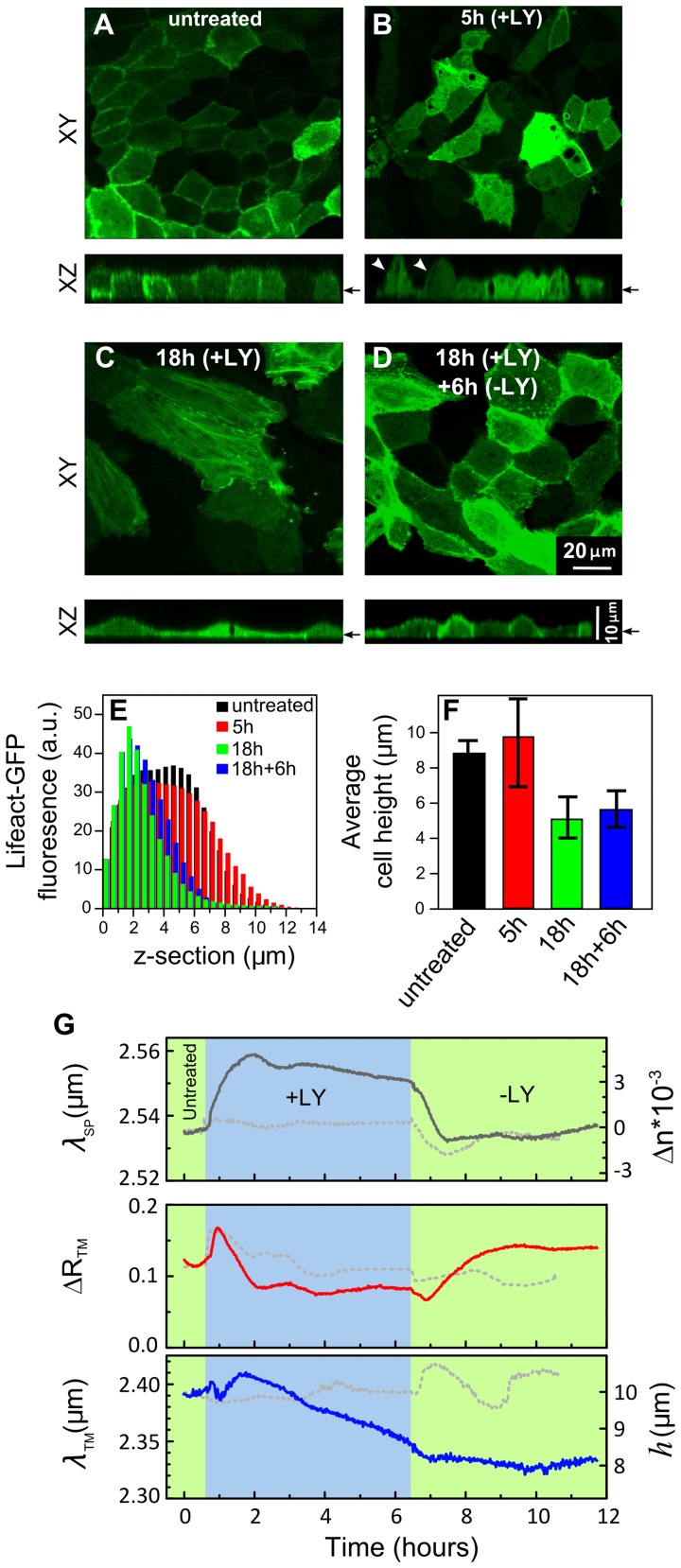
Effects of PI3K inhibition on cell monolayer height. A–D. Confocal images of live MDCK cells that stably express LifeAct-GFP. Cell monolayers were either untreated (a), or treated with LY for 5 hrs (b), or for 18 hrs (c), or treated with LY (+LY) for 18 hrs, then washed with plain growth medium and incubated with growth medium lacking LY for 6 hrs (−LY) to allow cell recovery (d). The confocal XY sections are taken at the level pointed out by arrows in the corresponding XZ sections. E. A histogram plot showing Lifeact-GFP fluorescence intensity levels measured at different Z-sections of untreated and LY-treated cells. F. The cell monolayer height as determined from the fluorescence intensity histograms (see Figure S5). The cell height heterogeneity is represented by error bars. G. Time-resolved measurements of *λ_SP_*, Δ*R_TM_* and *λ_TM_*. The measurements commenced in plain growth medium (untreated; green). After 30 min, the inhibitor was introduced to the flow chamber for 6 hrs (+LY; blue). Thereafter, the inhibitor was washed out with plain growth medium and the cells were re-exposed to the same medium for additional 5 hrs (−LY; green). In the control experiments, the cells were exposed to the DMSO solvent (dashed line).

To investigate this effect in live cells, we exposed live GFP-LifeAct- expressing MDCK monolayers to LY treatment and examined their morphology using confocal microscope (Figures 5A–D). The cell height was measured as described (see Figures 5E,F and S5). Prior to treatment, the cells in the monolayer revealed a typical cortical actin filament labeling and their height was approximately 8 µm (Figures 5A and F). The initial 5 hrs of cell treatment resulted in a significant heterogeneity in cell height (Figures 5B and F); some cells have bulged out of the cell layer (indicated with arrows, Figure 5B), thus exhibiting a taller morphology compared to the neighboring cells. Interestingly, after 18 hrs post-treatment, the cell monolayer remained confluent, yet, individual cells within the monolayer had considerably flattened, and their height appeared to be consistently reduced throughout the entire monolayer (Figures 5C and F). After drug removal and cell monolayer exposure to a plain growth medium for 6 hrs, the cells’ height and cortical actin filament labeling partially recovered (Figures 5D and F).

Next, we applied LY treatment to non-labeled MDCK cell monolayer cultured on gold substrate and structural changes were monitored using the infrared waveguide spectroscopy technique. The TM_1_ resonant wavelength *λ_TM_* was gradually blue-shifted during 6 hrs of LY treatment (+LY; Figure 5G, lower panel), indicating that cell height was reduced from 10 to 8 µm. The decrease in cell height slowed down significantly upon LY removal, and partially reversed after 3.5 hrs (−LY; Figure 5G, lower panel). The surface plasmon wavelength yields complementary information on cell layer morphology. Indeed, *λ_SP_* became red-shifted upon exposure to LY, indicating that the refractive index *n_eff_* nearby the substrate was increased, suggesting that the cell-substrate interactions became even more intimate in the LY-treated cells. Interestingly, following ∼1 hour of LY removal (−LY; Figure 5G, top panel) the shift in *λ_SP_* was completely reversed, essentially reaching its pretreatment values.

The slight reduction in Δ*R*_TM_ in response to LY treatment was similar to that obtained by the control treatment with DMSO solvent without LY. We believe that in both cases the Δ*R*_TM_ reduction was most probably related to the increased heterogeneity in cell heights (see Figure S5) rather than to cell monolayer disruption. This interpretation is also supported by the fact that *λ_SP_* was red-shifted during the LY treatment. However, the recovery in Δ*R_TM_* upon LY removal seems to be specific, suggesting that homogeneity in cell height is restored.

## Discussion

We demonstrate that tightly packed live epithelial cell monolayers can guide electromagnetic radiation. The infrared wavelengths are favorable for the waveguide mode excitation in a living cell layer for the following reasons: a) the typical thickness of an epithelial cell monolayer (*h*∼10 µm) is on the order of the infrared wavelength, and b) scattering losses on intracellular compartments are relatively low in that wavelength range [20]. Using different epithelial cell lines, we found that waveguide modes can be excited in (i) intact cell clusters of at least 100 µm in length; (ii) cell height uniformity of Δ*h* <1 µm.

In this study we show that waveguide mode spectroscopy can be used as a novel label-free method for real-time sensing of cell monolayer intactness and height. Similar properties can be monitored by confocal microscopy or atomic force microscopy (AFM). However, these methods can perform precise single cell analyses, and they cannot track with the same prescription a large cell population. In addition, AFM has a poor temporal resolution [6], [7]. In contrast, the infrared waveguide mode spectroscopy enables label-free monitoring of large cell populations (∼10^6^ cells) with submicron precision in cell height and a time resolution of seconds. Thus, this method can also be used to study the collective behavior of cells in the context of tissues and organs, and in response to various physiological and patho-physiological processes, such as epithelial-to-mesenchymal transition [27], the development of certain epithelial cancers [28], inflammatory bowel diseases [29], epithelial cell infection by microbial pathogens [30], and epithelial tissue injury [31].

An interesting issue arising from our research of the cell layer waveguiding phenomenon is the spectrum of thermal radiation that is emitted from warm blooded (∼300°K) organisms typically covered by skin epithelium. Since the epithelium layer can carry the waveguide modes that appear as dips in the infrared absorption spectra, we suggest that equivalent features may appear in the thermal emission spectra of these organisms, whose emission spectra spans from ∼5 to 40 µm. These features could be informative for diagnosing the thermal characteristics of tissues in disease states [32]–[34].

In summary, for the first time we have demonstrated an exciting biophotonic phenomenon of infrared light waveguiding in live epithelial cell monolayers. We applied this finding for monitoring the intactness and thickness of the epithelial cell layers. We believe that the demonstrated capacity of monitoring these cellular parameters in real-time, label free and with high sensitivity may pave the way for waveguide spectroscopy to become an attractive diagnostic tool in the future.

## Materials and Methods

### Infrared Optical Setup

Our experimental setup is based on the commercial FTIR spectrometer (Equinox 55, Bruker Inc.) equipped with a home-made surface-plasmon plug-in. The infrared beam is collimated, polarized and reflected from the right-angle ZnS prism [40×20 mm^2^ base (ISP Optics, Inc., Irvington, N.Y, US)] coated with 18 nm thick gold film (Figure 1A). The reflected beam is collected by the liquid-nitrogen-cooled MCT (HgCdTe) detector. In time-dependent measurements, reflectivity of *p*-polarized beam at fixed incident angle was normalized to the reflectivity of the *s*-polarized beam taken at time zero. Each spectral measurement takes ∼25 sec and represents an average over 8 scans with 8 cm^−1^ resolution.

### Data Processing

The monolayer cell height was calculated from the *TM*_1_ resonant wavelength, using the refractive index of the cell layer found from the wavelength of the surface plasmon resonance. To this end we used Fresnel equations for a four-layer assembly (ZnS/18 nm Au/cell layer/culture medium), to fit the measured reflectivity spectrum at known angle *θ_inc_* see Figure S2.

### Cell Preparation and Culture

MDCK cells were routinely cultured in growth medium (MEM Earle’s salts supplemented with 5% fetal bovine serum (FBS) and 1% antibiotics), as described [35]. Following three days in culture, a fully confluent cell monolayer was detached from the dish by trypsin treatment (0.25% Trypsin/EDTA in Puck’s saline A; Biological Industries, Israel). Cells were washed with plain growth medium and resuspended in 10 ml of the same medium. Then, 1.5 ml of the cell suspension was placed on the surface of a gold-coated prism, previously mounted on a sterile polycarbonate base. The cells were allowed to adhere to the Au surface for 30 min in the CO_2_ incubator (5% CO_2_, 37°C, 90% humidity). Then, 9 ml of growth medium were added gently to the cells, and the prism with cells was further incubated for 3–4 days in the CO_2_ atmosphere until a continuous cell monolayer was formed. The prism with cells was then attached to the flow chamber (Figure 2A), and cells were maintained under continuous flow (5 µl/min) of the fresh culture medium supplemented with 10 mM Hepes, pH 7.5. Cell treatment with media containing different Ca^2+^ concentrations, or the PI3K specific inhibitor LY294002, or DMSO was applied by introducing the relevant medium to the flow chamber at a high speed (200 µl/min) for 15 min, and subsequently at a slower rate (5 µl/min) for the remaining time.

### Ca^2+^ Depletion and Replenishment

Cell exposure to low Ca^2+^ concentration was achieved by exposing the cells to custom-made DMEM/F12 low-Ca^2+^ medium [36], [37] (Biological Industries, Beit Haemek, Israel) supplemented with 10 mM Hepes, pH 7.5. Ca^2+^ replenishment was achieved by bathing the cells with the same medium supplemented with 2 mM Ca^2+^.

### PI3K Inhibition with LY294002

The PI3K specific inhibitor, LY294002, was purchased from Calbiochem (Merck KGaA, Darmstadt, Germany) and stored as a 10 mM stock in DMSO at −20°C. Cells were treated with 50 µM LY, a concentration shown previously to inhibit effectively the kinase in MDCK cells [15].

### Optical Microscopy

Optical time-lapsed imaging of the cells cultured on the gold-coated prism surface was synchronized with the FTIR scans. The images were taken through a 0.5 mm thick optical window by a CMOS camera [Lw 575, Lumenera] connected to the high magnification optical zoom lens [NAVITAR 12X Zoom] using a halogen-lamp upright co-axial illumination.

### Confocal Microscopy

MDCK cells (∼10^5^ cells/ml) stably expressing LifeAct-GFP were cultured on Glass Bottom Culture dishes (14 mm microwell; MatTek, Co., MA, USA) and kept in CO_2_ incubator (5% CO_2_, 37°C; 95% humidity) for 4 days. Images of live cells were acquired by an Olympus FV-1000 confocal microscope equipped with an on-scope incubator (Life Image Services, Basel, Switzerland), which controls temperature and humidity, and provides an atmosphere with 5% CO_2_. Confocal images of MDCK cells expressing LifeAct-GFP (excitation at 485 nm; emission at 538 nm) were acquired from several locations in the monolayer, using a 40X/NA = 1.3 immersion objective. The average cell height for at least 20 cells was calculated using the ImageJ software [Rasband, W.S., ImageJ, U. S. National Institutes of Health, Bethesda, Maryland, USA, http://rsb.info.nih.gov/ij/, 1997–2012], as described in Figure S5.

## Supporting Information

Figure S1 **Infrared reflectivity spectra from the Au-coated substrate without cells.** Angular-resolved reflectivity spectra from a cell-free Au-coated ZnS prism. A ZnS prism was coated with an 18 nm thick Au film and covered with cell culture medium. Measurements were performed at exactly the same conditions as those in which we studied the excitation of waveguide mode in living cells, The only resonant feature is the strong reflectivity minimum (deep blue) arising from the surface plasmon resonance. Its angular dependence mimics the dispersion of the water refractive index. The results show that waveguide modes do not appear in the absence of a cell layer.
(TIFF)

Figure S2 **Modeling of reflectivity spectrum with Fresnel quad-layer model.** Experimental data of infrared reflectivity spectrum of an MDCK cell monolayer compared with the calculated spectrum of a four layer assembly (ZnS/18 nm Au-film/cell layer/culture medium this is confusing because in your theoretical background you considered a three-layer assembly). The following parameters have been used in the Fresnel simulation: internal incidence angle, *θ_inc_* = 34.6°, cell layer thickness *h* = 5.5 µm. The refractive index of each layer was determined by an independent measurement [20].
(TIFF)

Figure S3 **Waveguide modes in different epithelial cell monolayers.** A–H. Infrared reflectivity spectra from different cells cultures on an Au-substrate. Both surface plasmon (deep resonances) and waveguide modes (shallow resonances) are present, the latter are zoomed in panels B,D,F. The wavelength of the surface plasmon (SP) and waveguide modes (TM) could be fine-tuned by changing the incident angle, *θ_inc_*. The pale red color corresponds to a larger incident angle. Panels h and g exemplify the TM and SP resonances at longer wavelengths (*λ* = 3.5–4 µm).
(TIFF)

Figure S4 **Evolution of the surface plasmon resonance wavelength and depth resolves different phases in the process of MDCK cell monolayer formation.** A. Resonant wavelength of the surface plasmon (*λ_SP_*, upper panel) and corresponding refractive index change, Δ*n_d_* (right y-axis). The reflectivity at surface plasmon resonance (*R_min_*, lower panel) is determined by the losses on SP propagation. At *λ*∼2.5 µm the major contribution to the SP losses comes from the scattering of the SP wave on cell-medium interfaces, see [18]. B. *R_min_* as function of the cell coverage, as calculated from the *λ_SP_*, resolves different phases in cellular morphology; I- deposition and spreading of individual cells (concomitant growth of the cell coverage and perimeter of cell-covered regions), II- cell-cell attachment (growth in cell coverage and almost constant cell perimeter), III- monolayer closure (growth in cell coverage and decrease of cell perimeter).
(TIFF)

Figure S5 **Determination of cell height and its heterogeneity within a cell monolayer by the quantitative confocal imaging.** A. Confocal optical sectioning of live MDCK cells stably expressing LifeAct-GFP. The entire cell volume was visualized following fluorescent tagging of the actin cytoskeleton and thin (∼0.4 µm) confocal sectioning from below the substrate-cell interface (z = −0.5 µm), where fluorescent levels were minimal, up to the cells’ most apex regions where the recorded fluorescence levels became minimal (z = 13.0 µm). Montage of XY images taken at 0.5 µm intervals is shown. Bar = 50 µm. B. Determination of cell height (*h*). The average fluorescence intensity of LifeAct-GFP was measured at different *z*-sections using confocal microscopy. The background fluorescence level from the substrate plane (*z* = −0.5 µm) was subtracted and fluorescence levels were normalized to the maximum value. Cell height (*h*) was defined as the size where fluorescence level is above 20% from the maximum. The heterogeneity of the cell height (Δ*h*, i.e. intralayer variability) was taken as a half of width where the fluorescence drops from 80% to 20%. Note that floating or swollen cells which have bulged out of the cell layer (pointed by the red arrow-heads, plane A.) were excluded from the fluorescence intensity calculations.
(TIFF)
