# Correction: Real-Time Sensing of Cell Morphology by Infrared Waveguide Spectroscopy

**DOI:** 10.1371/annotation/a606531a-7eae-416e-86d6-74b9b413f2d8

**Published:** 2013-05-09

**Authors:** Victor Yashunsky, Tal Marciano, Vladislav Lirtsman, Michael Golosovsky, Dan Davidov, Benjamin Aroeti

There were errors in the Funding statement and Author Contributions.

The correct version of the Funding is: This work was supported by the NOFAR grant of the Israel Ministry of Industry and Trade. V.L. acknowledges support from the Lady Davis Fellowship Foundation. B.A. acknowledges support of the Israel Science Foundation (Grant 1167/08) and of the Israel Cancer Association. The funders had no role in study design, data collection and analysis, decision to publish, or preparation of the manuscript.

The correct version of the Author Contributions are: Conceived and designed the experiments: VY TM MG BA. Performed the experiments: VY TM. Analyzed the data: VY TM MG. Contributed reagents/materials/analysis tools: VY VL MG BA. Wrote the paper: VY TM VL MG BA DD. Conceived and supervised the project: MG BA DD. 

