# Supplementary figures and images for: Real-Time Sensing of Cell Morphology by Infrared Waveguide Spectroscopy
Source: PLoS One. 2012 Oct 31;7(10):e48454. doi: 10.1371/journal.pone.0048454 (PMC3485211; doi:10.1371/journal.pone.0048454)

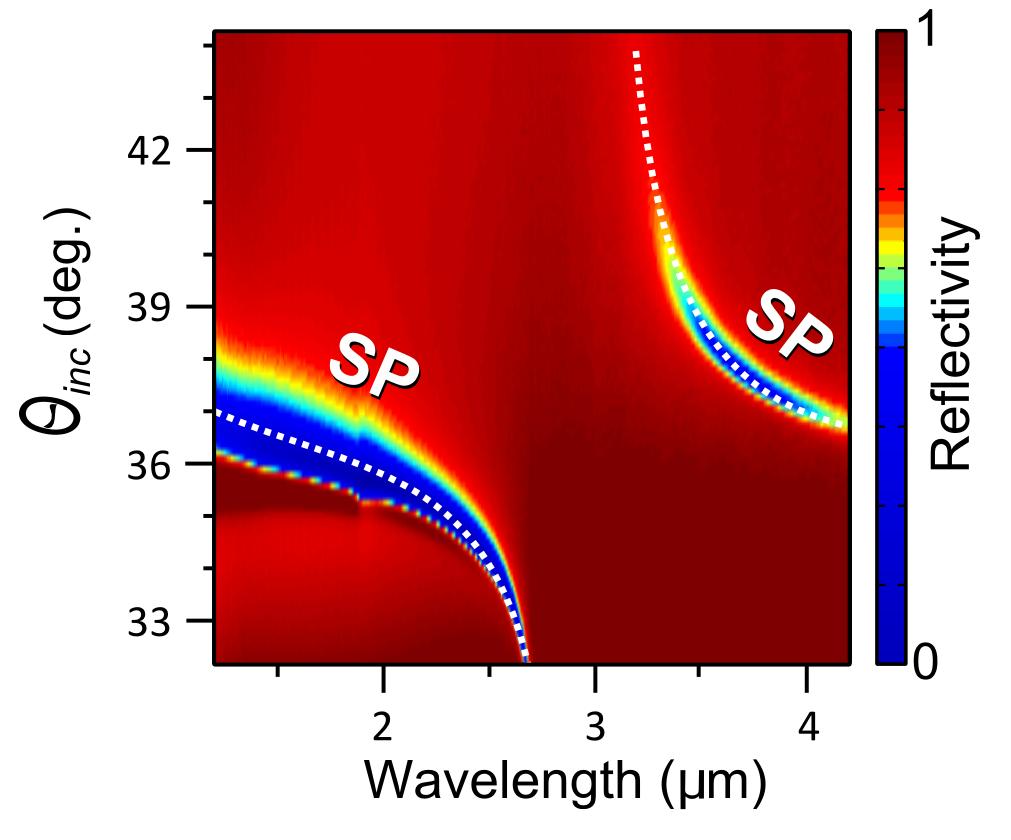

Supplement: Figure S1 — Infrared reflectivity spectra from the Au-coated substrate without cells. Angular-resolved reflectivity spectra from a cell-free Au-coated ZnS prism. A ZnS prism was coated with an 18 nm thick Au film and covered with cell culture medium. Measurements were performed at exactly the same conditions as those in which we studied the excitation of waveguide mode in living cells, The only resonant feature is the strong reflectivity minimum (deep blue) arising from the surface plasmon resonance. Its angular dependence mimics the dispersion of the water refractive index. The results show that waveguide modes do not appear in the absence of a cell layer. (TIFF) [file pone.0048454.s001.tiff]

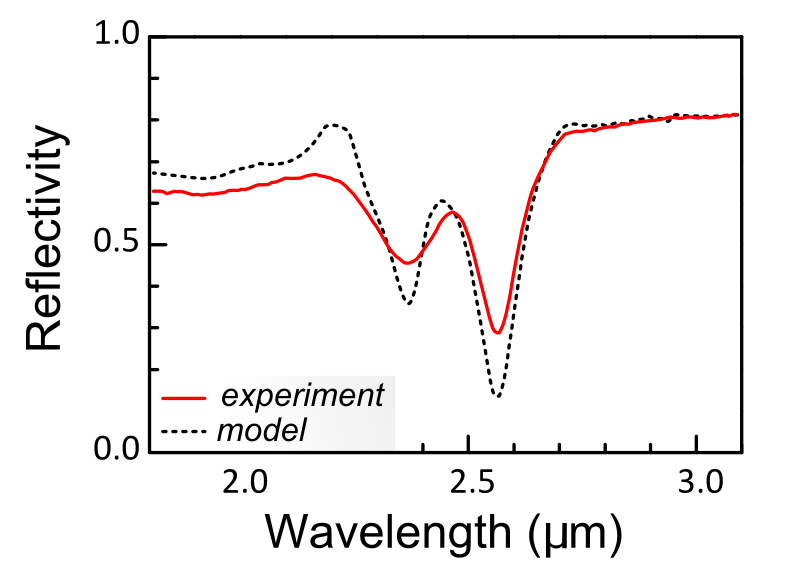

Supplement: Figure S2 — Modeling of reflectivity spectrum with Fresnel quad-layer model. Experimental data of infrared reflectivity spectrum of an MDCK cell monolayer compared with the calculated spectrum of a four layer assembly (ZnS/18 nm Au-film/cell layer/culture medium this is confusing because in your theoretical background you considered a three-layer assembly). The following parameters have been used in the Fresnel simulation: internal incidence angle, θinc = 34.6°, cell layer thickness h = 5.5 µm. The refractive index of each layer was determined by an independent measurement [20]. (TIFF) [file pone.0048454.s002.tiff]

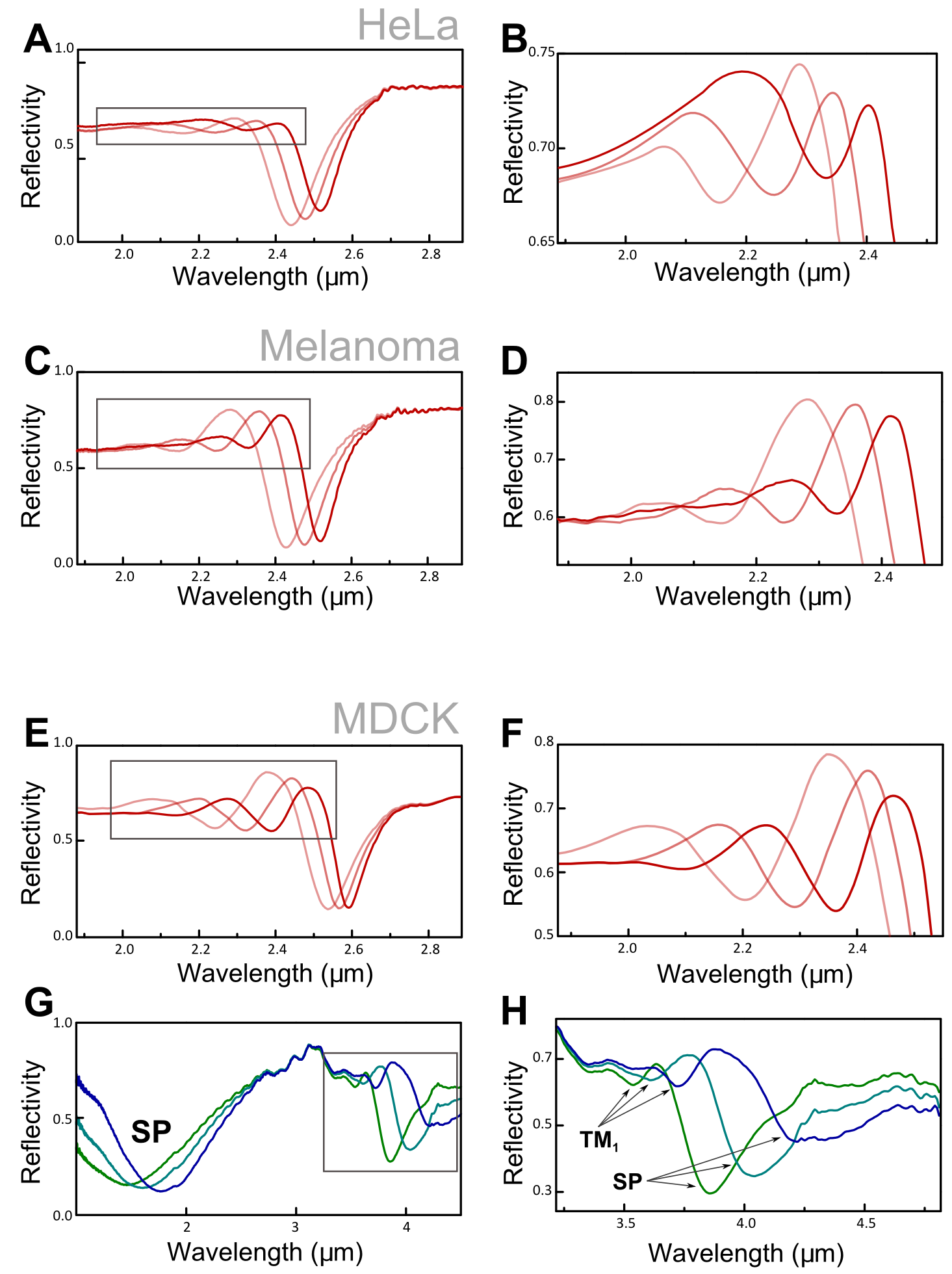

Supplement: Figure S3 — Waveguide modes in different epithelial cell monolayers. A–H. Infrared reflectivity spectra from different cells cultures on an Au-substrate. Both surface plasmon (deep resonances) and waveguide modes (shallow resonances) are present, the latter are zoomed in panels B,D,F. The wavelength of the surface plasmon (SP) and waveguide modes (TM) could be fine-tuned by changing the incident angle, θinc. The pale red color corresponds to a larger incident angle. Panels h and g exemplify the TM and SP resonances at longer wavelengths (λ = 3.5–4 µm). (TIFF) [file pone.0048454.s003.tiff]

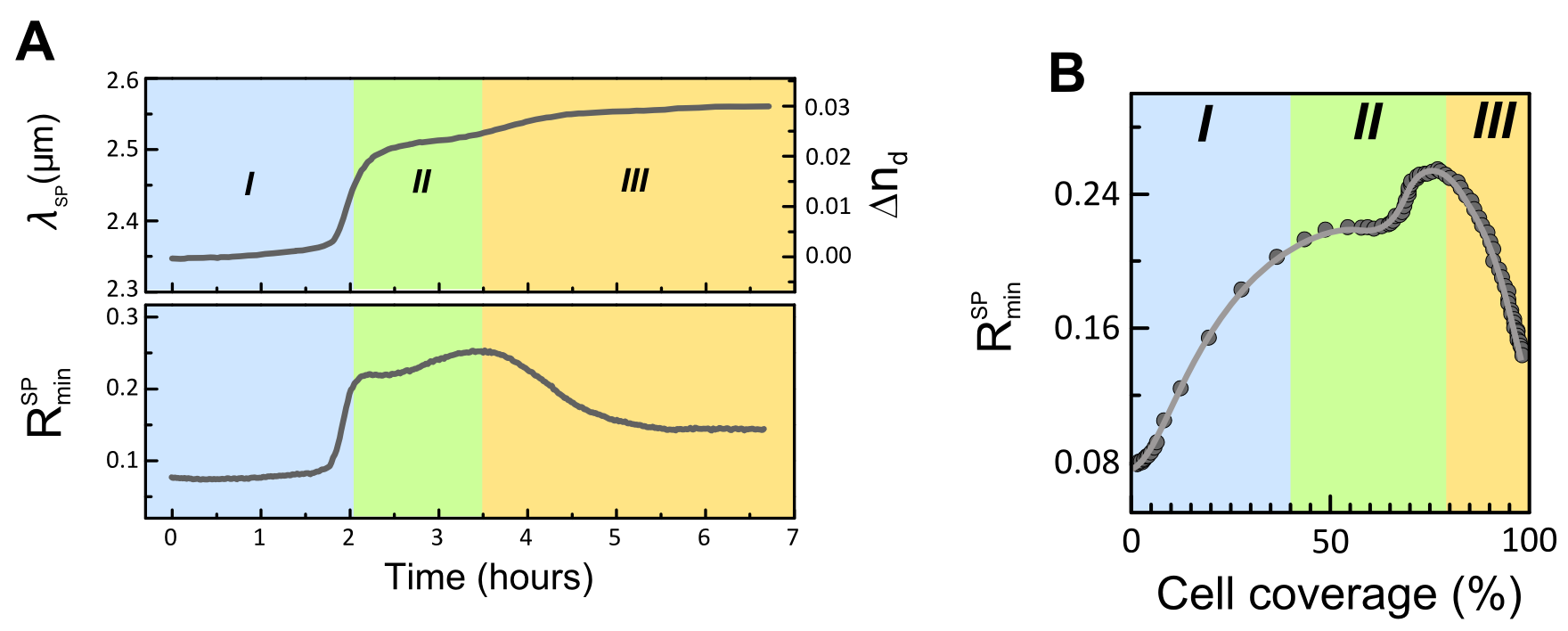

Supplement: Figure S4 — Evolution of the surface plasmon resonance wavelength and depth resolves different phases in the process of MDCK cell monolayer formation. A. Resonant wavelength of the surface plasmon (λSP, upper panel) and corresponding refractive index change, Δnd (right y-axis). The reflectivity at surface plasmon resonance (Rmin, lower panel) is determined by the losses on SP propagation. At λ∼2.5 µm the major contribution to the SP losses comes from the scattering of the SP wave on cell-medium interfaces, see [18]. B. Rmin as function of the cell coverage, as calculated from the λSP, resolves different phases in cellular morphology; I- deposition and spreading of individual cells (concomitant growth of the cell coverage and perimeter of cell-covered regions), II- cell-cell attachment (growth in cell coverage and almost constant cell perimeter), III- monolayer closure (growth in cell coverage and decrease of cell perimeter). (TIFF) [file pone.0048454.s004.tiff]

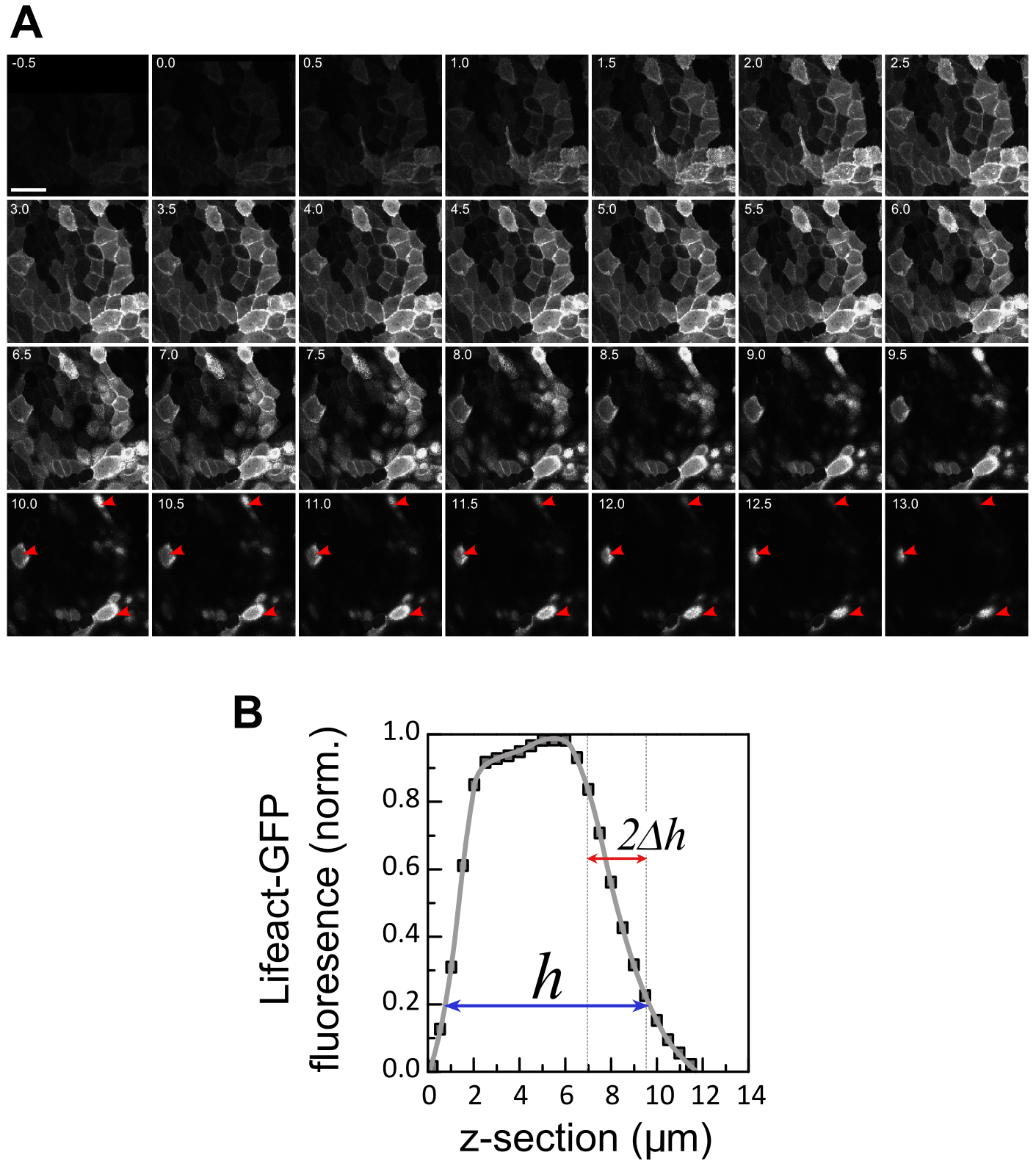

Supplement: Figure S5 — Determination of cell height and its heterogeneity within a cell monolayer by the quantitative confocal imaging. A. Confocal optical sectioning of live MDCK cells stably expressing LifeAct-GFP. The entire cell volume was visualized following fluorescent tagging of the actin cytoskeleton and thin (∼0.4 µm) confocal sectioning from below the substrate-cell interface (z = −0.5 µm), where fluorescent levels were minimal, up to the cells’ most apex regions where the recorded fluorescence levels became minimal (z = 13.0 µm). Montage of XY images taken at 0.5 µm intervals is shown. Bar = 50 µm. B. Determination of cell height (h). The average fluorescence intensity of LifeAct-GFP was measured at different z-sections using confocal microscopy. The background fluorescence level from the substrate plane (z = −0.5 µm) was subtracted and fluorescence levels were normalized to the maximum value. Cell height (h) was defined as the size where fluorescence level is above 20% from the maximum. The heterogeneity of the cell height (Δh, i.e. intralayer variability) was taken as a half of width where the fluorescence drops from 80% to 20%. Note that floating or swollen cells which have bulged out of the cell layer (pointed by the red arrow-heads, plane A.) were excluded from the fluorescence intensity calculations. (TIFF) [file pone.0048454.s005.tiff]
